# Supplementary material for: Content-rich biological network constructed by mining PubMed abstracts
Source: BMC Bioinformatics. 2004 Oct 8;5:147. doi: 10.1186/1471-2105-5-147 (PMC528731; doi:10.1186/1471-2105-5-147)
Supplement: Additional File 2 — The original results of the above study (non-essential files are deleted to keep the file size under the limit set by BMC bioinformatics). [file 1471-2105-5-147-S2.bz2 › chilibotAdditionalFile2/dip05/569ID10525310E10935/html/FGFR4_FGF19.html]

 


 **FGFR4** and **FGF19** 
  
Found 3 abstracts in PubMed, retrieved 3.  
 

 What does Google say? 
 PDF only 
| .edu only 

---

**Interactive relationship** (e.g. stimulation, inhibition, etc)

**Inhibitory relationship**- FGF 19  [ **FGF19** ]  also stimulated MAPK activation, an effect that was abrogated by a soluble dominant negative dn form of  **FGFR4** .  Ref: 12169442 Am J Physiol Endocrinol Metab, 2002
**Neutral relationship**- We conclude that  **FGFR4**  mediates FGF 19  [ **FGF19** ]  signaling to the PRL promoter.  Ref: 12169442 Am J Physiol Endocrinol Metab, 2002
- We show that FGF 19  [ **FGF19** ] , which selectively binds  **FGFR4** , can induce prolactin PRL but not growth hormone expression.  Ref: 12169442 Am J Physiol Endocrinol Metab, 2002
- FGF 19  [ **FGF19** ]  is a high affinity, heparin dependent ligand for  **FGFR4**  and is the first member of the FGF family to show exclusive binding to  **FGFR4** .  Ref: 10525310 Cytokine, 1999

**Non-interactive relationship** (e.g. studied together, co-existance, homology, etc.)

- In contrast, Ets enhanced FGF 19  [ **FGF19** ]  induced PRL activation but failed to confer an effect for FGF 19  [ **FGF19** ]  on the  **FGFR4**  promoter.  Ref: 12169442 Am J Physiol Endocrinol Metab, 2002
- FGF 19  [ **FGF19** ] , a novel fibroblast growth factor with unique specificity for  **FGFR4** .  Ref: 10525310 Cytokine, 1999
- We demonstrate that FXR directly regulates expression of fibroblast growth factor 19  [ **FGF19** ]  FGF 19  [ **FGF19** ] , a secreted growth factor that signals through the  **FGFR4**  cell surface receptor tyrosine kinase.  Ref: 12815072 Genes Dev< MedlineTA>Genes Dev, 1999
- Thus, we examined whether FGF 19  [ **FGF19** ]  might regulate its own receptor through the Ets Ik element in the  **FGFR4**  promoter.  Ref: 12169442 Am J Physiol Endocrinol Metab, 2002
